# High-resolution non-contrast free-breathing coronary cardiovascular magnetic resonance angiography for detection of coronary artery disease: validation against invasive coronary angiography

**DOI:** 10.1186/s12968-022-00858-0

**Published:** 2022-04-11

**Authors:** Muhummad Sohaib Nazir, Aurélien Bustin, Reza Hajhosseiny, Momina Yazdani, Matthew Ryan, Vittoria Vergani, Radhouene Neji, Karl P. Kunze, Edward Nicol, Pier Giorgio Masci, Divaka Perera, Sven Plein, Amedeo Chiribiri, René Botnar, Claudia Prieto

**Affiliations:** 1 School of Biomedical Engineering and Imaging Sciences, King’s College London, St Thomas’ Hospital, 4th Floor Lambeth Wing, Westminster Bridge Road, London, SW1 7EH UK; 2 British Heart Foundation Centre of Excellence and National Institute for Health Research Biomedical Research Centre at the School of Cardiovascular Medicine and Sciences, Kings College London, London, UK; 3 Department of Biomedical Imaging Science, Leeds Institute of Cardiovascular and Metabolic Medicine, University of Leeds, Leeds, UK; 4 MR Research Collaborations, Siemens Healthcare Limited, Frimley, UK; 5 Royal Brompton Hospital, Guy’s and St Thomas Hospital NHS Trust, London, UK; 6 Escuela de Ingeniería, Pontificia Universidad Católica de Chile, Santiago, Chile

**Keywords:** Coronary imaging, Cardiovascular magnetic resonance angiography, Coronary artery disease

## Abstract

**Background:**

Coronary artery disease (CAD) is the single most common cause of death worldwide. Recent technological developments with coronary cardiovascular magnetic resonance angiography (CCMRA) allow high-resolution free-breathing imaging of the coronary arteries at submillimeter resolution without contrast in a predictable scan time of ~ 10 min. The objective of this study was to determine the diagnostic accuracy of high-resolution CCMRA for CAD detection against the gold standard of invasive coronary angiography (ICA).

**Methods:**

Forty-five patients (15 female, 62 ± 10 years) with suspected CAD underwent sub-millimeter-resolution (0.6 mm^3^) non-contrast CCMRA at 1.5T in this prospective clinical study from 2019–2020. Prior to CCMR, patients were given an intravenous beta blockers to optimize heart rate control and sublingual glyceryl trinitrate to promote coronary vasodilation. Obstructive CAD was defined by lesions with ≥ 50% stenosis by quantitative coronary angiography on ICA.

**Results:**

The mean duration of image acquisition was 10.4 ± 2.1 min. On a per patient analysis, the sensitivity, specificity, positive predictive value and negative predictive value (95% confidence intervals) were 95% (75–100), 54% (36–71), 60% (42–75) and 93% (70–100), respectively. On a per vessel analysis the sensitivity, specificity, positive predictive value and negative predictive value (95% confidence intervals) were 80% (63–91), 83% (77–88), 49% (36–63) and 95% (90–98), respectively.

**Conclusion:**

As an important step towards clinical translation, we demonstrated a good diagnostic accuracy for CAD detection using high-resolution CCMRA, with high sensitivity and negative predictive value. The positive predictive value is moderate, and combination with CMR stress perfusion may improve the diagnostic accuracy. Future multicenter evaluation is now required.

**Supplementary Information:**

The online version contains supplementary material available at 10.1186/s12968-022-00858-0.

## Introduction

Coronary artery disease (CAD) remains the single most common cause of death worldwide. Imaging can be used to assess CAD, which has gained increasing importance given the low yield of CAD identification during routine invasive coronary angiography (ICA) [1]. Coronary computed tomography angiography (CCTA) is the non-invasive reference standard for assessment of anatomical CAD, by means of excellent spatial and temporal resolution that enables sharp delineation of the coronary arteries. The vast diagnostic and prognostic evidence of CCTA have seen it mature into clinical guidelines for routine assessment of CAD [2, 3].

Coronary cardiovascular magnetic resonance angiography (CCMRA) is an attractive alternative to CCTA derive anatomical detail of the coronary arteries [4], which may be advantageous in selected patients as it does not require administration of iodinated contrast or use of ionizing radiation. Furthermore, CMRA may allow simultaneous soft tissue characterization and does not suffer from blooming artefact in heavily diseased coronary arteries, which can make luminal assessment challenging in patients with advanced atheroma [5]. Traditionally, routine implementation of CCMRA into clinical practice has been hampered by long and unpredictable acquisition times, suboptimal spatial resolution and significant patient motion which may degrade image quality. Recently developed methods have been developed to achieve submillimeter resolution CCMRA within a short timeframe with advanced motion correction of non-rigid respiratory-induced motion of the heart and 100% respiratory scan efficiency, which may thereby facilitate implementation into a routine clinical workflow [6, 7].

More recently, this technique shows good diagnostic accuracy against CCTA [8], although the prevalence of CAD was relatively low. Thus, the objective of this study was to prospectively determine the diagnostic accuracy of CCMRA against the gold standard of ICA in patients with suspected CAD.

## Methods

### Study population

consecutive patients with suspected CAD scheduled for ICA as part of routine clinical care were recruited to this prospective single center study between 2019—2020. Patients were recruited either before (n = 24) or after (n = 21) ICA. For the latter, patients were only included if no revascularization was undertaken in the interval. Exclusion criteria were contraindication to CMR, previous coronary artery bypass graft (CABG) surgery, previous coronary stents, atrial fibrillation or unstable angina. The study was approved by the National Research Ethics Service (15/NW/0778) and written informed consent was obtained from all participants.

### CMR protocol

CMR scans were performed on a 1.5 T CMR  scanner (MAGNETOM Aera, Siemens Healthineers, Erlangen, Germany) with a dedicated 32-channel spine coil and an 18-channel body coil.

#### Patient preparation

In order to promote coronary vasodilatation and control heart rate, patients were given sublingual 800 mcg glyceryl trinitrate and intravenous metoprolol (Betaloc ®, AstraZeneca, United Kingdom), titrated in 5 mg aliquots to target a heart rate of 60 bpm.

#### Image acquisition

A multi-slice survey was performed in the axial, coronal and sagittal planes. A free-breathing 4 chamber cine was acquired using a balanced steady state free precession (bSSFP) sequence, to ascertain the optimal time period during which there was the least cardiac motion, typically the diastolic phase of the cardiac cycle. This acquisition was performed free-breathing to mimic the same conditions of the free-breathing CCMRA acquisition, since breath holds during image acquisition have been shown to alter heart rate [9]. Typical parameters for the cine acquisition included: echo time 1.16 ms, repetition time 2.32 ms, flip angle 50°, voxel size 1.8 × 1.8× 6 mm, segmented acquisition, retrospective gating and 25 phases per cardiac cycle.

The CCMRA acquisition used an electrocardiogram (ECG)-triggered undersampled (threefold acceleration factor) free-breathing 3D whole-heart, bSSFP sequence with a 3D variable density spiral-like Cartesian trajectory with golden-angle rotation, as previously described [7]. Typical parameters included: echo time 1.6 ms, repetition time 3.7 ms, flip angle 90°, bandwidth per pixel 890 Hz and field of view 320 × 320 × 86–115 mm. A low-resolution 2D image-navigator (iNAV) preceded each spiral-like interleave which allows for 100% respiratory scan efficiency, predictable scan time and 2D translational motion correction of the heart on a beat-to-beat basis [10]. A spectrally selective spectral presaturation with inversion recovery (SPIR) fat saturation pulse with a constant flip angle of 130° was used to improve coronary delineation and minimize fat-related artefacts. An adiabatic T2 preparation module (40 ms) was used at each heartbeat in order to enhance the contrast between blood and cardiac muscle, and thereby avoid extracellular contrast agents. The reconstructed voxel size was 0.6 mm^3^ (acquired isotropic resolution of 0.9 mm^3^). Acquisition times were recorded.

#### Motion corrected image reconstruction

This consisted of three main steps as previously described [8], which involves beat-to-beat respiratory binning and intra-bin translational motion correction using 2D iNAV [10], bin-to-bin 3D non-rigid motion correction [11] and 3D patch-based low-rank reconstruction [7].

### Image analysis

CCMRA images were analyzed after 3D multiplanar reformatting using Osirix (Pixmeo SARL, Bernex, Switzerland) by two expert readers in both CMR and CCTA with greater than 5 years of experience (PGM and MSN). The presence and degree of CAD was determined based on a visual analysis of DICOM images using a nine segment coronary segmentation model [12]. Analysis was performed on a per-patient level (at least one coronary artery with stenosis ≥ 50%) and per-vessel level. The process was repeated in 10 random cases, six months later to assess observer variation.

Image quality was graded on a per-patient and per-vessel analysis on a 4-point scale (1 = poor, 2 = average, 3 = good and 4 = excellent). The diagnostic quality was determined for each segment (0 = non diagnostic, 1 = diagnostic).

### Coronary angiography

Patients underwent ICA at St Thomas’ Hospital, London. Quantitative coronary angiography (QCA) was performed retrospectively on x-ray coronary angiography images offline (Medcon Ltd., Tel Aviv, Israel) by an experienced observer with more than 5 years of experience in coronary angiography. Obstructive CAD was defined by a stenosis in a major artery of ≥ 50% on QCA.

### Statistical analyses

Statistical analyses were performed using GraphPad Prism 8 software (version 8.4.1, GraphPad Software, San Diego, California, USA). Continuous variables were expressed as mean ± standard deviation or median and interquartile range according to the distribution. Normality distribution was assessed histograms and using Shapiro-Wilks test. Categorical variables are expressed as frequency (%). On a patient level, CAD was defined by the presence of at least one coronary artery with a lesion of ≥ 50% stenosis. On per territory basis, CAD was defined by presence of a vessel with at least one with stenosis of ≥ 50%. Diagnostic accuracies were calculated for sensitivity, specificity, positive predictive value and negative predictive value and the 95% confidence intervals were computed as previously described [13, 14]. Observer agreement was assessed with Cohen’s Kappa coefficient for categorical data. Two tailed values of p < 0.05 were considered statistically significant.

## Results

Two patients could not complete the scan due to claustrophobia, and one scan was excluded due to the presence of an intracoronary cardiac stent. Thus, the final analysis was composed of 45 patients. The prevalence of obstructive CAD was 42%. Median time interval between CCMRA and ICA was 11 (interquartile range 3-35) days. Mean duration of CCMRA imaging was 10.4 ± 2.1 min with 100% respiratory scan efficiency. Baseline patient demographics and cardiovascular risk factors are shown in Table 1 and distribution of CAD is shown in Table 2.

**Table 1:** Patient demographics and cardiovascular risk factors (n = 45)

Demographics	n=45
Age (years)	62 ± 10
Gender female	15F (33%)
Height (m)	1.71 ± 0.10
Weight (kg)	90 ± 19
Body mass index (kg/m^2^)	31 ± 6
Cardiovascular risk factors	
Hypertension	35 (78%)
Hypercholesterolemia	31 (69%)
Diabetes mellitus	15 (33%)
Smoking history	17 (38%)
Family history of coronary artery disease	21 (47%)
Previous myocardial infarction	5 (11%)
Hemodynamic data	
Heart rate during CMRA scan (beats per minute)	61 ± 8
Resting systolic blood pressure (mmHg)	126 ± 16
Resting diastolic blood pressure (mmHg)	73 ± 11

Data presented as n (%) and as mean ± standard deviation

**Table 2:** Distribution of disease as defined by invasive coronary angiography

Vessel	n (%)
LAD	13 (29)
LCx	9 (20)
RCA	7 (16)
LM	1 (2)
Distribution of disease	
Single vessel	11 (24)
Two vessel	6 (13)
Three vessel	2 (4)

Obstructive lesions were defined ≥ 50% stenosis on quantitative coronary angiography. *LAD* left anterior descending coronary artery, *LCx* left circumflex coronary artery, *LM* Left main coronary artery, *RCA* right coronary artery

### CMRA image quality

On a per-patient analysis, median score for CCMRA image quality was 3 (interquartile range 2–3). On a per-vessel analysis, median image quality score for the left anterior descending artery (LAD): 3 (interquartile range 2–3), left circumflex (LCx): 3 (interquartile range 2–4) and right coronary artery (RCA): 3 (interquartile range 2–4). On a per segment analysis, diagnostic image quality was achieved in 100% of left main (LM) segment, 92% of LAD segments, 91% of LCx and 93% of RCA segments. Diagnostic image quality was achieved in 98% of proximal, 92% of mid and 87% of distal segments.

### Hemodynamic data

Mean dose of intravenous metoprolol administered was 12 ± 6 mg. Mean heart rate during image acquisition was 61 ± 8 beats per minute. Resting systolic blood pressure and diastolic blood pressure for patients were 126 ± 16 and 73 ± 11 mmHg, respectively.

### Diagnostic accuracy

On a per-patient analysis, the sensitivity, specificity, positive predictive value (PPV) and negative predictive value (NPV) (95% confidence intervals) were 95% (75–100), 54% (36–71), 60% (42–75) and 93% (70–100), respectively.

On a per-vessel analysis the sensitivity, specificity, PPV and NPV (95% confidence intervals) were 80% (63–91), 83% (77–88), 49% (36–63) and 95% (90–98), respectively.

There was a good interobserver agreement from the two reporters (κ = 0.74, p = 0.02).

**Fig. 1.**
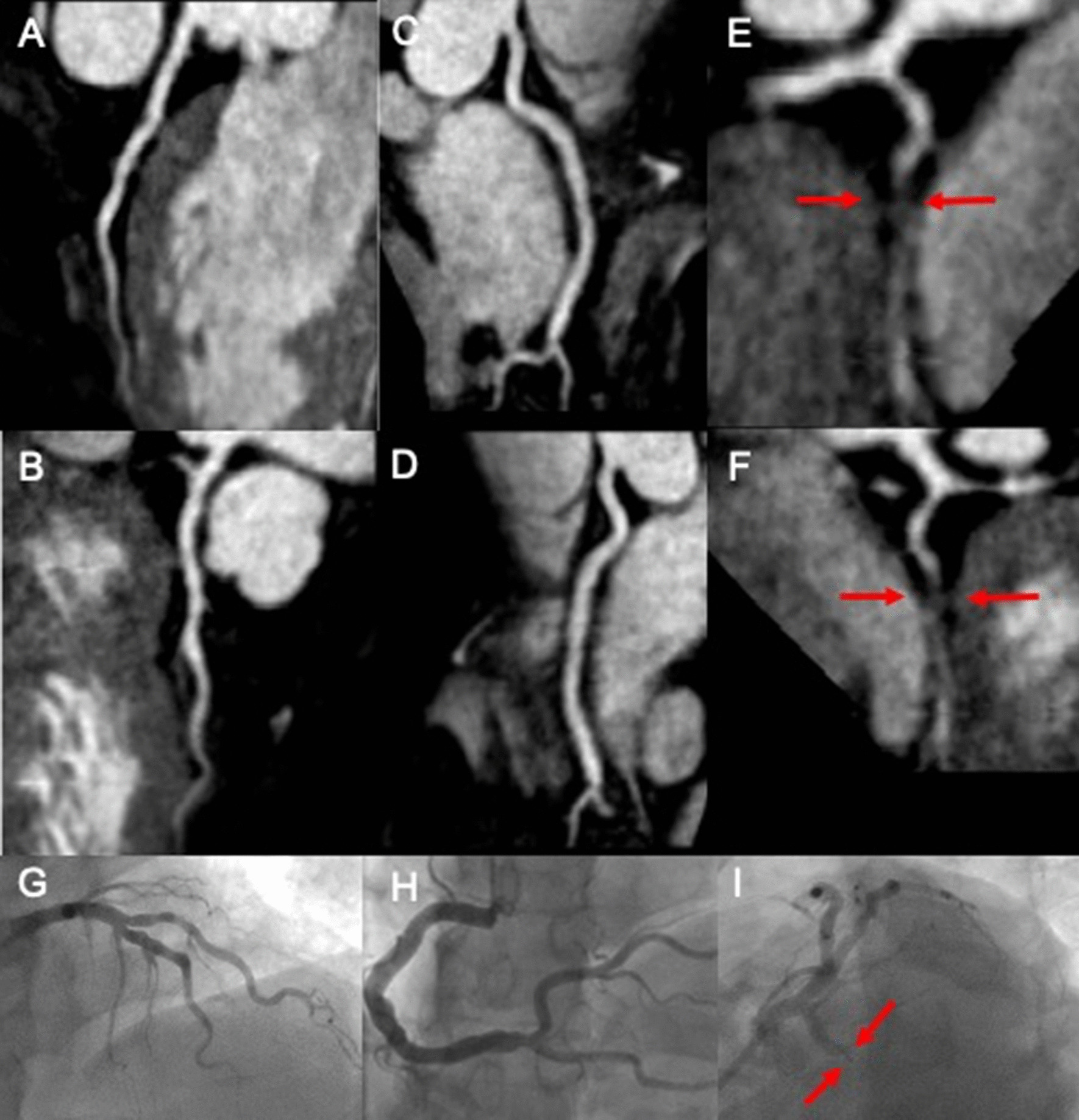
3D curved multi-planar reformats of a coronary cardiovascular magnetic resonance angiography (CCMRA) in a patient with chest pain and a history of hypertension, hypercholesterolemia and a family history of coronary artery disease. There is no significant disease in the left anterior descending coronary artery (LAD) **A**, **B** or right coronary artery (RCA) (**C**, **D**). However, there is an occluded left circumflex artery coronary artery (LCx), red arrows (**E**, **F**). These findings were confirmed during invasive coronary angiography (**G**–**I**, Additional file 1: Video S1)

**Fig. 2.**
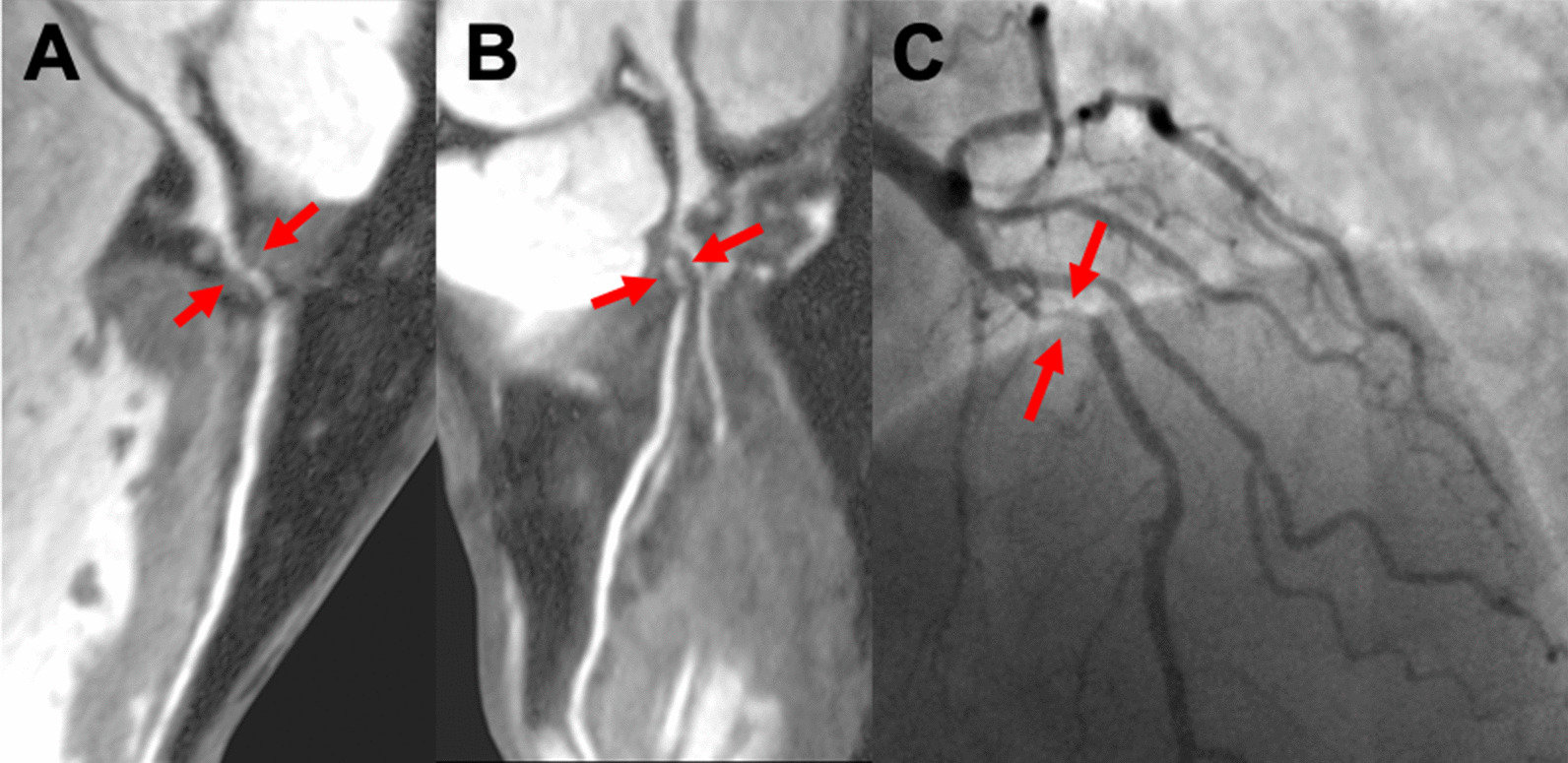
3D curved multi-planar reformat of a CCMRA in a male patient with exertional chest pain with a history of hypertension. There is an obstructive lesion (> 50%) in the LAD (Panel **A** and **B**, red arrows). These findings were confirmed during invasive coronary angiography (**C** and Additional file 2: Video S2)

**Fig. 3.**
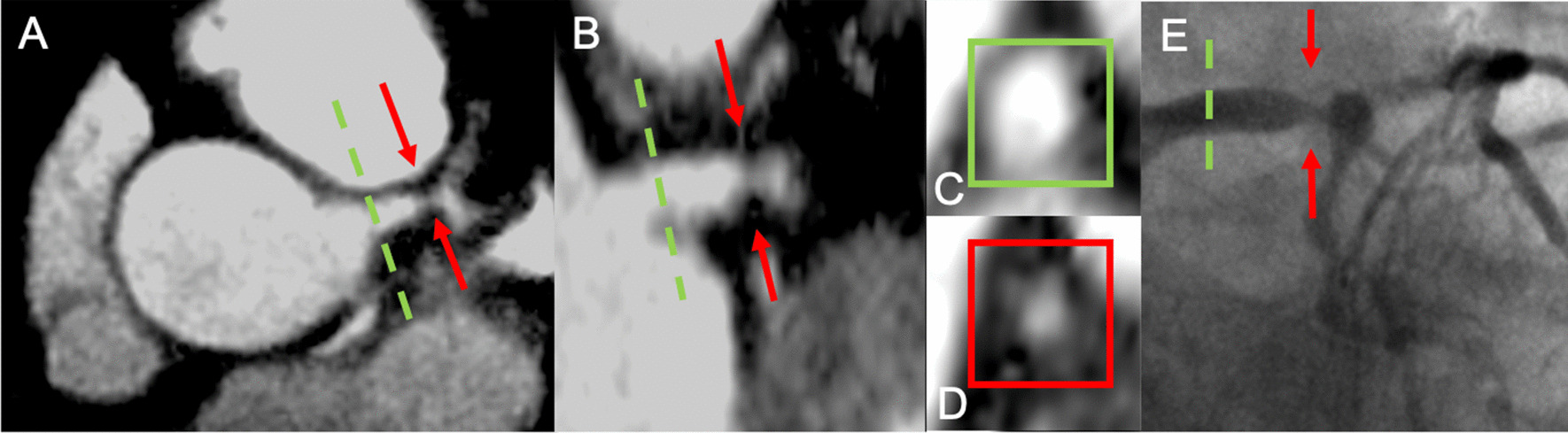
3D multi-planar reformat of a CCMRA in a patient with exertional chest pain on a background of hypertension and type 2 diabetes mellitus. Orthogonal views in Panel **A** and **B**, cross section views in Panel **C** and **D**. There is no significant disease in the proximal LM (green dashed line), but there is greater than 50% stenosis in the distal left main coronary artery (LM) (red arrow). These findings were confirmed during invasive coronary angiography (Panel **E** and Additional file 3: Video S3)

**Fig. 4.**
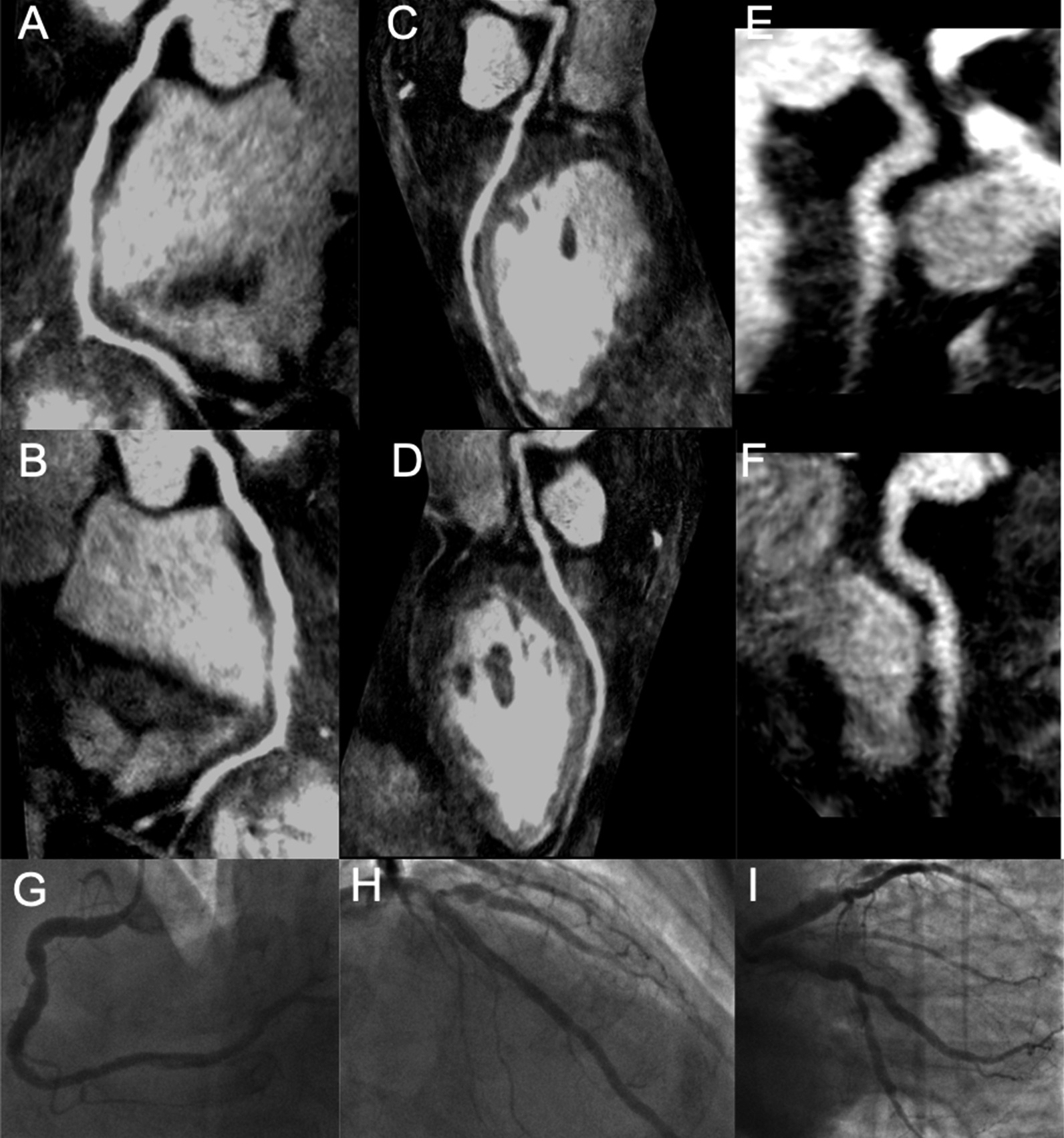
3D curved multi-planar reformats of a CCMRA in a patient investigated for suspected chest pain with no cardiovascular risk factors showed no obstructive disease. Large dominant RCA (**A**, **B**). LAD (**C**, **D**). Small non dominant LCx (**E**, **F**). These findings were confirmed during invasive coronary angiography (**G**–**I** and Additonal file 4: Video S4 and Additional file 5: Video S5)

The full breakdown of the diagnostic accuracies are presented in Table 3. Typical case examples are shown in Figs. 1, 2, 3 and 4 and Additional file 1: Videos S1, Additional file 2: Videos S2 and Additional file 3: Videos S3.

**Table 3:** Diagnostic accuracy of high-resolution coronary cardiovascular magnetic resonance angiography (CCMRA) for the detection of obstructive coronary artery disease defined by invasive coronary angiography

	Sensitivity (%)	Specificity (%)	Positive Predictive Value (%)	Negative Predictive Value (%)
Patient level	95 (75–100)	54 (36–71)	60 (42–75)	93 (70–100)
Territory level	80 (63–91)	83 (77–88)	49 (36–63)	95 (90–98)
LAD	92 (67–100)	69 (51–82)	55 (35–73)	96 (79–100)
LCx	67 (35–88)	81 (65–90)	46 (23–71)	91 (76–97)
RCA	86 (49–100)	79 (64–89)	43 (21–67)	97 (84–100)

Sensitivity, specificity, positive predictive value and negative predictive value are in %. 95% confidence intervals in brackets

## Discussion

Using non-contrast high-resolution CCMRA, we demonstrated a high diagnostic accuracy with high sensitivity and negative predictive value for detection of CAD compared to the invasive reference standard of ICA in a rapid timeframe of ~ 10 min.

In a preceding study [8], compared to reference standard of CCTA, there was a high sensitivity, specificity, PPV, NPV and diagnostic accuracy of 100% (95% CI: 76–100%), 74% (95% CI: 58–85%), 55% (95% CI: 35–73%), 100% (95% CI: 88–100%) and 80% (95% CI: 67–89%) respectively, for the detection of CAD. However, that study was performed in a cohort of patients with a lower risk of and prevalence of CAD, and the comparator was CCTA, rather than the invasive gold standard of ICA [7]. In this current study, we investigated patients with a comparatively higher prevalence of CAD, and we confirmed that this CCMRA technique has a high sensitivity and a high NPV, suggesting a potential role as a rule out test for CAD. The moderate PPV (60% and 49% at the patient and territory level respectively), may be due to a number of reasons. Firstly, the analysis of degree of stenosis was performed by readers in consensus, and despite blinding to the findings from the coronary angiogram, may have led to inadvertent overestimation of degree of stenosis, in order not to miss clinically relevant CAD lesions. Secondly, the intermediate PPV may relate to the inferior spatial resolution of CMRA (reconstructed to 0.6 mm^3^) compared to ICA (~ 0.2 mm), which may result in an overestimation in the degree of stenosis. Furthermore, we found that the diagnostic performance in the LCx territory was lower, which may be explained by the lower signal-to-noise ratio due to the greater distance from the coils from the LCx. Furthermore, the beat-to-beat anterior–posterior motion is not corrected for in this proposed approach, which may not correct the motion of the LCx in the lateral plane. Thus, future studies should investigate methods that enhance spatial resolution without compromising the total acquisition time, boosting signal to noise in the LCx and further improving motion correction.

The speed of acquisition of CMR clinical scans is highly relevant for clinical workflow, patient comfort and effective healthcare resource utilization. Often, CMR scans are considered as lengthy scans with variable scan times between patients, although technological developments such as parallel imaging, multichannel coils and compressed sensing have allowed more rapid clinical protocols [15–18]. The average time of acquisition of CCMRA in this study was approximately 10 min, which we feel is acceptable for routine integration for clinical workflow. For instance, the CCMRA acquisition could potentially be acquired after gadolinium contrast injection in a predictable scan time, by which time after 10 min, late gadolinium enhancement (LGE) could be acquired, as performed in routine clinical lists which require LGE imaging. The speed of acquisition has been achieved through the development of advanced motion correction frameworks that allow for 100% respiratory scan efficiency in combination with undersampled reconstruction. However, the acquisition time of CCMRA, typically acquired over several hundred heart beats, is still substantially longer in comparison to that of CCTA, which can be acquired in single heartbeat using modern technology. The longer image acquisition time is critically important, as any significant patient related movement cannot be easily corrected for.

CCMRA for the evaluation of CAD has been established over two decades ago [19, 20], and despite initial promise in multicenter evaluation [21], has not become part of routine clinical assessment in CMR. This may in part relate to the long unpredictable acquisition times and respiratory inefficiency from previous techniques. One recent study used compressed sensing to accelerate image acquisition within a rapid timeframe, although in that study, contrast administration was required [22], and did not compare to the invasive reference standard of ICA as we did in this study. More recently, obstructive CAD seen on CCMRA was found to provide incremental prognostic value over traditional risk factors [23]. One important step to clinical translation in our study is the ability to achieve 100% respiratory scan efficiency and high-resolution imaging with predictable scan times (thus not dependent on the specific subject’s breathing pattern). This is made possible by a 3D non-rigid motion corrected reconstruction framework with golden-step variable density spiral-like Cartesian trajectory with good undersampling properties which allows for 100% scan efficiency.

Whilst the focus of this study was to determine the degree of luminal stenosis compared to ICA, there has been much interest into plaque morphology. For example, there are established imaging biomarkers derived from CCTA which indicate adverse clinical outcomes such low attenuation plaque and positive remodeling [24] and perivascular fat [25]. Using computational fluid dynamics, non-invasive assessment of fractional flow reserve can be derived, which has shown to have high diagnostic accuracy and is associated with prognostic outcome data [26, 27]. There are also features that can be derived from CCMRA that indicate adverse clinical outcome, such as with high intensity plaques from T1-weighted images [28]. These features were not investigated as part of this current study, although the ability to determine the presence of vulnerable plaque and hemodynamic significance of lesions are likely to have incremental benefit beyond that of the degree of stenosis, although future studies are required to determine this.

The assessment of CAD is important for patients so that appropriate medical therapy can be initiated, adverse clinical outcomes can be prevented and revascularization considered for relief of angina symptoms [29]. The integration of high-resolution CMRA into a clinical workflow for assessment of CAD is particularly attractive given the multiparametric nature of CMR, which can be used to derive precise measurements of *left ventricular function*, *viability* (derived from regional motion wall abnormalities and LGE) and *ischemia* (derived from dynamic first pass perfusion imaging or dobutamine stress). The integration of *anatomy* as part of a multiparametric assessment has the potential to provide a complete assessment of CAD to guide clinical decision making and revascularization from a single exam. We feel that this is particularly important for clinical decision makers; whilst a negative stress perfusion scan is associated with a low risk of adverse outcome [30], from a perfusion scan alone, standard CMR does not derive information on coronary plaques and stenosis. Therefore, using standard CMR techniques may otherwise miss the opportunity to initiate preventative medical therapy based on the presence of coronary plaque with statins and alter clinical outcomes, as has been shown with CCTA [31]. Thus, the integration of high-resolution CCMRA may provide incremental diagnostic and prognostic value over standard CMR methods in patients with suspected CAD, and this will be the focus of our future research, which we hope will benefit our patients.

## Limitations

Firstly, this study was performed at a single center, using a single vendor at 1.5T field strength. Secondly, our analysis of CCMRA data was performed based on visual analysis rather than automated analysis of degree of stenosis. Nevertheless, the analysis was performed by two expert CMR readers, with good interobserver agreement. Thirdly, we excluded patients with previous CABG or coronary stents, and therefore the diagnostic performance in these patients is unknown, but is likely to be suboptimal due to the susceptibility artefact from stents and surgical clips. Forthly, we did not undertake intracoronary assessment with intravascular ultrasound or optical coherence tomography for the assessment of coronary artery lesions. Finally, we excluded patients with atrial fibrillation, as the variation of R-R interval would likely result in suboptimal image quality given variation in the acquisition period in the cardiac cycle.

## Conclusion

We demonstrate a high diagnostic accuracy of high-resolution non-contrast CCMRA within predictable scan time of ~ 10 min for the diagnosis of CAD. Future work is required through large multicenter evaluation for diagnostic accuracy and clinical outcomes in patients with CAD. CCMRA could be considered for routine integration in CMR protocols combined with stress perfusion and viability imaging, and in turn lead to a comprehensive assessment for the workup of patients with suspected CAD .

## Supplementary Information

**Additional file 1: Video S1.** Invasive s-ray coronary angiography confirms occluded proximal left circumflex coronary artery.

**Additional file 2: Video S2**. Invasive x-ray coronary angiography confirms severe stenosis in mid left anterior descending coronary artery.

**Additional file 3: Video S3**. Invasive x-ray coronary angiography confirms severe distal left main coronary arterydisease.

**Additional file 4: Video S4.** Invasive x-ray coronary angiography confirms no significant obstructive disease in the right coronary artery.

**Additional file 5: Video S5.** Invasive x-ray coronary angiography confirms no significant obstructive disease in the left coronary artery.

## Data Availability

The datasets used and/or analysed during the current study are available from the corresponding author on reasonable request. Dr. Debiao Li served as the *JCMR* Guest Editor for this manuscript.    Declarations

## Abbreviations

bSSFP: Balanced steady state free precession
CABG: Coronary artery bypass graft surgery
CAD: Coronary artery disease
CCMRA: Coronary cardiovascular magnetic resonance angiography
CCTA: Coronary computed tomography angiography
ECG: Electrocardiography
ICA: Invasive coronary angiography
iNAV: Image navigator
LAD: Left anterior descending coronary artery
LCx: Left circumflex coronary artery
LGE: Late gadolinium enhancement
LM: Left main coronary artery
NPV: Negative predictive value
PPV: Positive predictive value
QCA: Quantitative coronary angiography
RCA: Right coronary artery
SPIR: Spectral presaturation with inversion recovery
